# Hepatitis E Virus Genotype 1, Cuba

**DOI:** 10.3201/eid1408.080049

**Published:** 2008-08

**Authors:** María de la Caridad Montalvo Villalba, Licel de los Angeles Rodríguez Lay, Vivek Chandra, Marité Bello Corredor, Susel Sariego Frometa, Aidonis Gutierrez Moreno, Shahid Jameel

**Affiliations:** * Institute for Tropical Medicine “Pedro Kourí,” Havana, Cuba; † International Center for Genetic Engineering and Biotechnology, New Delhi, India

**Keywords:** Hepatitis, HEV, Cuba, letter

**To the Editor:** Hepatitis E virus (HEV) causes acute viral hepatitis, which in rare cases leads to fulminant hepatitis with high death rates, especially among women in their third trimester of pregnancy (*1*). Sporadic infections and epidemics have been reported from all parts of the world, especially Asia, Africa, and Latin America. Although indigenous hepatitis E has rarely been observed in industrialized countries, higher than expected anti-HEV prevalence has been detected in these areas (*1*). In the Caribbean region, many countries including Cuba, Haiti, Guatemala, and Honduras have reported hepatitis E (*2*,*3*), but the viruses have not been characterized.

The transmission of HEV is primarily fecal–oral, through contaminated drinking water; limited zoonotic transmission has also been reported. Despite only 1 serotype, 4 major genotypes of HEV have been reported (*1*). Genotype 1 is mainly responsible for sporadic infections and large outbreaks in Asia and Africa. Genotype 2 was first found in Mexico and later on the African continent. Genotypes 3 and 4 have been reported from the United States, Europe, China, Japan, and Taiwan; this group also includes the related swine HEV (*1*).

We report the phylogenetic analysis of 11 HEV isolates from 2 outbreaks and sporadic cases in Havana, Cuba. The first outbreak occurred in 1999 in a factory; 20 persons were affected (12 women, 8 men; median age 45 years, range 22–53 years). The second outbreak was in 2005 in a suburb of Havana and involved 26 persons (15 women, 11 men; median age 24 years, range 17–45 years). We also analyzed HEV in 12 sporadic clinical cases obtained from the Cuban national surveillance program for viral hepatitis. Most patients reported asthenia, epigastric pain, nausea, and vomiting. None had any history of international travel, contact with persons traveling from disease-endemic areas, or consumption of exotic foods. Serologic screening showed all patients to be negative for immunoglobulin (Ig) M against hepatitis A and hepatitis C viruses. One patient had positive results for hepatitis B surface antigen but negative results for anti-hepatitis B core antigen IgM and hepatitis B virus DNA. All patients were positive for anti-HEV IgM (Genelab Diagnostics, Singapore) according to the manufacture’s criteria. A total of 22 serum samples (outbreak 1, n = 9; outbreak 2, n = 7; sporadic cases, n = 6) were tested for HEV RNA; only 2 (both from sporadic cases) were positive. A total of 31 serum samples were also tested for anti-HEV IgG (Genelab Diagnostics), of which 22 were positive (outbreak 1, n = 7/10; outbreak 2, n = 10/13; sporadic cases, n = 5/8).

A total of 44 stool samples were collected 2–4 weeks after onset of symptoms and stored at –70°C until use. Fecal samples were screened for HEV open reading frame (ORF) 2 by using reverse transcription (RT)–PCR (*4*). For genotyping, nested RT-PCR was then performed for the ORF1 RdRp region (*5*) on 18 samples that were positive for ORF2. Of the 12 PCR products obtained, 11 fragments were cloned into pGEMT Easy Vector (Promega, Madison, WI, USA). At least 3 positive clones for each sample were sequenced, and the consensus sequence was used for phylogenetic analysis. The GenBank accession numbers of ORF1 for the HEV outbreak cases from Cuba are CUB10-1999 (EU165504), CUB11-1999 (EU165502), CUB13-1999 (EU1655019), CUB19-1999 (EU165500), CUB24-1999 (EU165499), CUB68-2005 (EU165496), and CUB71-2005 (EU165495). For the sporadic cases they are CUB9-2005 (EU165503), CUB1803-2003 (EU165494), CUB2-2005 (EF493155), and CUB27-2005 (EU165498). Additionally, nested RT-PCR was conducted with ORF2-specific primer pairs (*6*) for 2 HEV isolates, 1 each from outbreak and sporadic cases, and sequences were obtained. The accession numbers for ORF2 are outbreak CUB10D-1999 (EU284749) and sporadic CUB2D-2005 (EU284748).

Phylogenetic analysis of ORF1 nucleotide sequences showed that HEV isolates from Cuba clustered in genotype 1 with high bootstrap values (Figure, panel **A**). The same genotype was detected in an outbreak of hepatitis E in UN peacekeepers deployed from Bangladesh to Haiti (*3*). Although the outbreak was adequately contained, anti-HEV immunoglobulin was subsequently detected in 3% of civilians in Haiti (*3*). Nucleotide identity between isolates from Cuba and other HEV strains from genotype 1 ranged from 91.7% to 99%. The strains from Cuba were closely related to the isolates from India and shared 97.8%–99% homology with Yam-67 (*7*). Absolute ORF1 nucleotide differences (p-distances; MEGA2 software, www.megasoftware.net) of isolates from Cuba ranged from 0% to 1.6%, demonstrating a high degree of relatedness. The ORF2 analysis supported our ORF1 findings because the CUB2D-2005 and CUB10D-1999 sequences also clustered with genotype 1 (Figure, panel **B**). Both strains from Cuba shared 96.1% nucleotide homology with a prototype strain from Burma (Bur82) and were related to the strains from India (Hyderabad and Yam-67), sharing 97.4%–99% homology. Absolute ORF2 nucleotide differences ranged from 0.8% to 1.9%. This value for ORF1 ranged from 0.05% to 0.08% for the same isolates from Cuba (CUB2-2005 and CUB10-1999).

**Figure:**
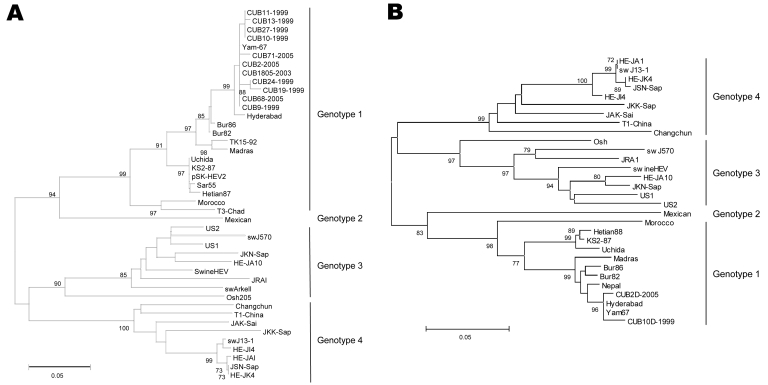
Phylogenetic trees constructed on the basis of A) 240 nucleotides, RdRp region, from open reading frame (ORF) 1, and B) 311 nucleotides from ORF2. Each tree was generated by using the neighbor-joining method; the distance matrix was calculated by using the Kimura 2-parameter method. The robustness of the trees was determined by bootstrap for 1,000 replicates. Values >70% are shown at the nodes. The major branches represent hepatitis E virus genotypes. Scale bar indicates 0.05 substitutions per nucleotide position.

HEV shows a global presence. The genotype distribution, although dominant in a given geographic area, is not limited to that area. For example, genotype 2, first identified on the American continent in Mexico (*8*), was later found in Namibia and Nigeria on the African continent (*9*,*10*). We report indigenous HEV genotype 1 strains in the Americas.
